# Cross-sectoral follow-up after hospital discharge from a geriatric ward: a study protocol of a randomised controlled trial (G-UD)

**DOI:** 10.1186/s13063-025-08922-7

**Published:** 2025-07-01

**Authors:** Alexander Viktor Eriksen, Sanne Have Beck, Dorthe Nielsen, Katja Thomsen, Josefine Oredson Krone, Karen Andersen-Ranberg

**Affiliations:** 1 Geriatric Research Unit, Department of Clinical Research, University of Southern Denmark, Odense, Denmark; 2 Department of Geriatric Medicine, Odense University Hospital, J.B. Winsløwsvej 4, Odense C, 5000 Denmark; 3 Danish Aging Research Centre, Department of Public Health, University of Southern Denmark, Odense, Denmark

**Keywords:** Geriatrics, Post-discharge follow-up, Readmission, Randomised controlled trial, Acute readmission, Older adults, Community-dwelling, Home care, Transitional care management, Discharge

## Abstract

**Background:**

Advancing age is associated with multimorbidity, polypharmacy and functional impairments and older adults with these characteristics are at increased risk of acute admission and readmission. Readmission rate of geriatric patients is high, especially within the first week after discharge. Previous studies on transitional care interventions have mainly showed positive effects, but no study has assessed the effect of a home visit done jointly by a geriatric nurse and a community nurse, with a systematic review of health functions, objective clinical assessment and bedside blood analyses.

**Methods:**

A single-centre two-arm parallel group randomised controlled trial using permuted block randomisation will be conducted. Randomisation occurs at discharge. The intervention consists of a follow-up visit within 2–5 days by a geriatric nurse and a community nurse. A systematic review of bodily functions is carried out, and solutions of identified problems are discussed taking into consideration the patient’s view. If the patient shows signs of worsened health since discharge vital status and bedside blood analyses using point-of-care-testing (POCT) can be carried out at the discretion of the geriatric nurse. As required the patients will be conferred with a senior geriatrician by video consultation. The patient’s primary care physician is invited to participate online. The control group will receive usual discharge. Endpoints are readmission, mortality rates and financial costs. User perspectives include focus group interviews with patients, geriatric nurses, community nurses, geriatricians and primary care physicians.

**Discussion:**

The intervention is multifactorial to match the needs of complex geriatric patients. We will thus not be able to discern, which part of the intervention has the greatest impact, but the intervention will encompass most of the clinical situation that may potentially lead to acute readmission. If a positive effect is found the intervention may be scaled up to include all departments discharging vulnerable geriatric patients, as well as implemented in guidelines for the discharge of older medical patients.

**Trial registration:**

ClinicalTrials.gov, Identifier: NCT05139823, Registered 1 December 2021.

**Supplementary Information:**

The online version contains supplementary material available at 10.1186/s13063-025-08922-7.

## Background

Geriatric patients are characterised by high age, multimorbidity and polypharmacy, frequently in combination with functional impairments [1, 2]. This makes them frail and particularly vulnerable to an increased risk of acute hospital admissions and readmissions [3, 4]. Despite the ongoing demographic change towards more old and oldest olds [5, 6], the number of hospital beds has been reduced markedly [7], mainly achieved by reducing in-hospital length of stay (LOS) [8]. At the Department of Geriatrics, Odense University Hospital (G-OUH) LOS declined from 11 days in 2006 to 6 days in 2023 (personal communication). Consequently, older patients are discharged in a more vulnerable state than previously, but with care support delivered by the municipal home care.

In Denmark, readmissions occur in about 10% of 65 + year old [9] but increases with higher age [10]. The risk of readmission increases with the number of comorbidities, medications and prior contacts to the primary health care providers [10], all being characteristics of the geriatric patient.

For geriatric patients admitted to G-OUH, readmission occurs in 19% of discharged patients according to hospital administrative data, and the median number of days until readmission is nine [11]. Most patients are readmitted with a diagnosis unrelated to their index admission, with pneumonia and dehydration being the most common causes—even when these were not the primary diagnoses at the time of the index admission.[11].

Several intervention studies aiming at reducing acute readmission by post-discharge follow-up exist [12]. A recent systematic review of transitional care interventions consisting of both pre- and post-discharge components found that transitional care interventions reduce readmissions among older medical patients [13]. However, the review includes several older studies as well as studies from countries not comparable to Denmark due to differences in the health care systems, and the overall conclusion is that high-quality studies are lacking [13]. In Denmark, two randomised controlled studies examining acute 30-day readmission among geriatric patients, i.e., non-disease-specific multimorbid older adults with functional loss have emerged within the last 3 years [14, 15], which both showed significant reductions in acute readmissions after post-discharge follow-up. However, one of the studies was quasi-randomised and included geriatric patients discharged from the emergency department [14]. The other study included patients discharged from a geriatric department, but the ward staff was non-blinded to intervention allocation [15]. Furthermore, in this study the intervention was carried out by hospital staff, i.e., a geriatrician and a nurse or physiotherapist, while the home care was invited to join, but not as a necessity.

A recent before-and-after study in geriatric patients discharged to a municipal intermediate care facility has shown that post-discharge follow-up visits by a team consisting of a specialist physician in geriatric medicine and a geriatric nurse reduce acute 30-day re-admission by 28% [16]. Qualitative data from interviews of staff from the same study indicates that the geriatric team visit gives the municipal nurses at the intermediate care facility a feeling of self-confidence and feeling of increased competencies when working interdependently [17]. This supports the concept of a face-to-face cross-sectoral exchange of information on health and care in the presence of the newly discharged patient is important to cover all aspects of health recovery.

The number of health professionals dedicated to older patients does not balance the growing number of diseased older people [18]. Digital technology offers a possibility for alleviating the challenges of scarce personnel resources. Geriatricians may function as virtual consultants by video [19], thereby supporting experienced geriatric nurses with competences in doing objective clinical assessment as well as collecting and analysing biological samples on-site. The increased digitisation in health care together with the development of various bed-side point-of-care testing (POCT) technologies, e.g., biochemical analyses, ultrasound, has increased the possibilities for more efficient and high-quality health services without jeopardising the safety of older vulnerable patients. There is an urgent need to examine the effect, as well as the challenges, in using digital health services for clinical decision-making on discharged geriatric patients during the first vulnerable days at home after discharge. We hypothesise that the in-person meeting between a geriatric hospital nurse and a community nurse, who works closely with the municipal home carers can reduce acute readmission and other challenges related to the transition by early identification of health-related problems arising immediately post-discharge.

In this article, we present the protocol for a planned RCT, which will be conducted to examine an intervention that helps alleviating the challenges related to transition from a specialised acute hospital ward to the geriatric patient’s home and personal care provided by the municipal home care. We present our study, with a thorough description of our methods.

## Methods

This paper follows the SPIRIT 2013 statement [20]. No large language models or other AI has been used in the creation of this manuscript.

### Aim

The aim of this study is to investigate the effects of a coordinated and simultaneous home visit 2–5 days after discharge, by a community nurse and a geriatric nurse equipped with POCT equipment on 30-day readmissions.

### Study design

The study is a single-centre two-arm parallel group randomised controlled trial with an aim to show superiority. It will utilise permuted block randomisation without stratification. Distribution will be equivalent between the two groups. The study will be conducted over approximately 24 months.

### Setting and study population

The study will take place at the Department of Geriatrics Odense University Hospital Region of Southern Denmark (G-OUH) and in the Odense Municipality, Denmark.

Odense Municipality is by population the largest municipality in the Region of Southern Denmark and the 4th largest municipality in Denmark [21]. The population (2022) is approximately 206,000 [21], of which approximately 36,000 are 65 + years of age [21]. Odense Municipality has 14,000 employees [22], including 3500 employees caring for frail older adults and disabled persons [23]. This includes both care at home and public care homes. The mean life expectancy in Odense Municipality is 81.2 years (2018–2022) [24]. In 2021, 5500 citizens received home care [25]. The municipal home care is extensive and covers housing for older citizens, cleaning, personal care, help taking medication, physical training, provision of aids and appliances and help carrying out activities of daily living. The municipal home care works 24/7 and delivers care at home based on a needs assessment. The municipality also provides 1123 care home dwellings (i.e. “1123 dwellings” in care homes), not including dwellings in private care homes (*n* = 449). Furthermore, home care service is allocated to the individual citizen based on a needs assessment, and irrespective of the presence of a cohabitating partner. If a family member provides care for a relative, they can apply for respite or relief [26].

The municipality employs social and health care workers as well as nurses (community nurses; home-based-care nurses) to provide care and nursing care. All citizens are allocated a primary care physician, who serves as the gatekeeper to the hospital. The Danish health care system offers a universal health care coverage financed through taxes [27].

Odense University Hospital is a medium-sized university hospital situated in Odense municipality. It has 965 beds, 95,000 annual discharges and approximately 315,000 patient-days of admissions per annum. The hospital provides 412 of 652 highly specialised functions and approximately 10,000 employees [28]. Odense University Hospital primarily covers the island of Funen, Denmark, but has also many highly specialised functions offered both as regional functions and at a national level. It is the largest hospital in the Region of Southern Denmark and the second largest hospital in Denmark by number of beds.

The Department of Geriatric Medicine at Odense University Hospital is a specialised medical department with geriatric specialist consultants caring for up to 32 in-hospital beds and a large outpatient clinic. In 2018, there were 2232 unique patients discharged from the ward. The mean age was 82.4 years, and the mean LOS was 6.1 days. The Department of Geriatric Medicine at Odense University Hospital receives acutely admitted geriatric patients, which are characterised by medical complexity, high age, multimorbidity, polypharmacy and functional loss. Medically non-complex patient with a simple single disorder (e.g. otherwise healthy adult with pneumonia) are allocated to any specialised internal medicine ward but may also be allocated to G-OUH according to availability of beds. However, due to the vast number of older medically complex patients, simple single disorder patients are few at G-OUH. The most common primary discharge diagnoses of patients discharged from G-OUH are acute infections and dehydration, but with considerable overlap with a variety of other common diagnoses, e.g. falls, rhabdomyolysis, heart failure, chronic obstructive pulmonary disease (COPD) and delirium.

All G-OUH patients are initially assessed in the acute medical unit (AMU). Patients are either referred to the AMU by their primary care physician or a doctor on call or may have called the emergency services. Upon arrival in the AMU, they are seen by AMU physicians. The acute medical unit physicians making the initial assessment are specialists of acute medicine or specialists of any internal medicine speciality (gastroenterology, rheumatology, pulmonology, endocrinology, infectious diseases or geriatric medicine). Patients admitted with cardiac symptoms are initially assessed by a physician from cardiology before going to either the AMU or the cardiology ward. Patients allocated to G-OUH are transferred to the geriatric ward if total LOS is expected to be greater than 48 h. This is determined in the AMU by either a geriatric team (physician, physiotherapist, occupational therapist and nurse) during daytime or by the AMU physician on duty during evening or night. If the LOS is expected to be less than 48 h for a patient allocated to G-OUH, the patient will be treated in the AMU by the geriatric team, but not physically moved to the ward. If continued intravenous treatment is necessary, the geriatric team can discharge the patient to their home, where the treatment can be continued and monitored by a municipal acute home care team equipped with point of care blood sampling. Occasionally, patients expected with a shorter stay are transferred to G-OUH, due to crowding in the AMU.

### Eligibility

A project coordinator identifies eligible patients among all patients admitted to G-OUH.

Only patients transferred to G-OUH ward are screened for eligibility, and thus patients discharged directly from the AMU are not included.

Criteria for eligibility are patients admitted to G-OUH and discharged to their own home and in need of personal care provided by the municipal home care organisation in Odense Municipality. Patients discharged to care home are also included. Exclusion criteria are patients discharged to other skilled nursing facilities or discharged with no need for personal care. Also, terminally ill patients and incapacitated patients, e.g., patients with moderate or severe dementia are not eligible. Patients with dementia are excluded if they are unable to live independently (i.e., if they require a care home or are completely dependent on their spouses to remain at home). In cases of uncertainty, a senior geriatric consultant will assess the cognitive function and determine severity. Patients that are delirious throughout the entire hospital stay will also be excluded, as they cannot give informed consent. Patients with active delirium are reassessed by a project manager based on indications such as confusion to orientation (to personal data, treatment, location, time and recent events), ability to concentrate and follow a conversation, organisation and coherence of thought, and overall mental status, as assessed by the CAM score. In cases of uncertainty, patients are not included. Finally, while initially excluding readmitted patients who had already been enrolled as study participants, it was decided after 4.5 months from the start of the study to allow the inclusion of readmitted study participants. The change was made due to an insufficient entry of eligible participants following a shortage of staff and a cut in the number of hospital beds at G-OUH. The original eligibility criteria were thus slightly changed to allow readmitted patients to participate. We decided to include these patients due to the uncertainty of future patient flow, knowing that we can adjust for this factor in the analyses after the trial.

### Recruitment and enrolment

**Fig. 1:**
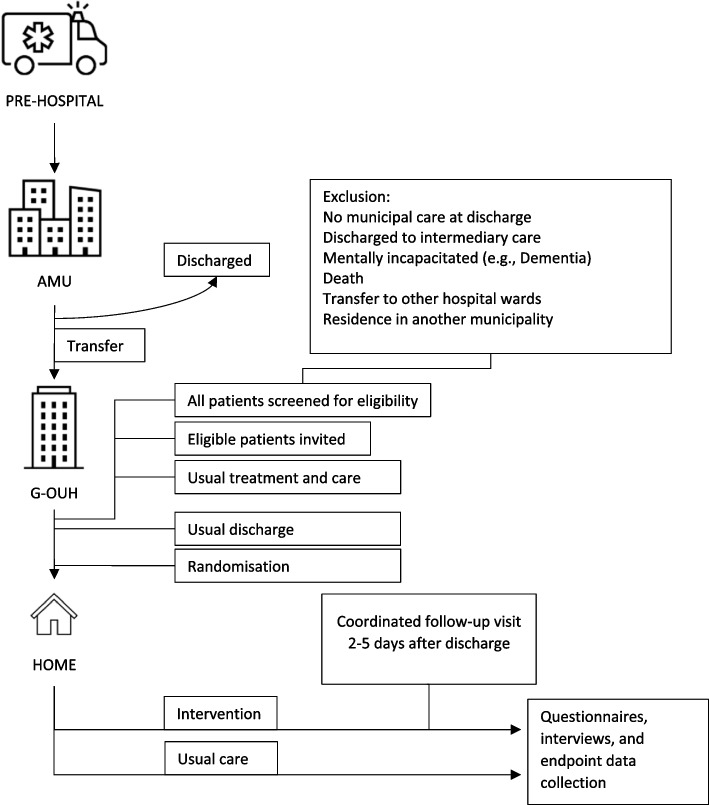
Study design

During weekdays, patients transferred to G-OUH ward are screened for eligibility by a project coordinator in accordance with the criteria described above. Eligible patients are invited as soon as possible. Patients transferred to G-OUH during weekends are screened for eligibility on the first weekday. Patients that are hospitalised late Friday or Saturday and discharged during the weekend are likely missed (Table 1, Fig. 1).

**Table 1:** SPIRIT—Schedule of enrolment, allocation, assessments, intervention, and follow-up

**Time point**	**Enrolment** **-** ***t***_**1**_		**Allocation**	**Intervention**	**Follow-up**			
		Admission	Discharge	Home-visit 2–5 days	30 days	90 days	180 days	1 year
Enrolment								
Eligibility screening	X							
Informed consent	X							
Demographic data		X						
Data collection								
Barthel-100 ADL Index		X						
Clinical Frailty Scale			X					
Multimorbidity			X					
Polypharmacy			X					
Charlson Comorbidity Index			X					
Vital parameters			X	X				
Length of Stay			X					
Discharge diagnoses			X					
FS3 Nursing Problem Areas			X	X				
Discharge Questionnaire			X					
Post-Discharge Questionnaire					X			
Allocation			X					
Intervention Planning			X					
Intervention				X				
POCT Measurements				X				
Follow-up data								
Mortality data					X	X	X	X
Readmission data					X	X	X	X

Eligible patients are informed about the project (orally and in writing) and invited to join. If they accept, they sign an informed consent. They are randomised on the day of discharge to either the intervention or control group. In the case of a planned discharge during the weekend, the participants are randomised on Friday. Participants randomised to the control group receive a usual discharge, while participants in the intervention group receive a usual discharge as well as a coordinated follow-up visit, which is planned to take place 2–5 working days after the day of discharge.

For all participants (control and intervention), administrative and clinical data will be collected from hospital records and the hospital administrative system, e.g. hospital discharge diagnoses, LOS, medication, medical history, Charlson Comorbidity Index [29] and Barthel Activities of Daily Living (ADL) score.

### Intervention and usual care

#### All participants

All participants giving informed consent receive the usual care, i.e., diagnostics, medical treatment and care by geriatricians, geriatric nurses and therapists. The usual discharge procedure is as follows: When a patient is close to not needing daily medical assessment by a ward geriatrician, a discharge date is set. A geriatric nurse communicates digitally with the municipal home care about the patient’s care needs in the home setting, including information about the patient’s in-hospital needs of personal care, need for aids and appliances and help with medication. The information also includes an overview of the in-hospital treatment. The patient is not discharged until the municipal home care is ready to provide the patient with the required help for personal care as jointly decided by the hospital staff and the municipal home care. In the case of increased home care requirements compared to before admission, the municipal home care is given a 48-h notice before discharge. If there is no increase in the level of home care, the notice is given 24 h before discharge. Due to these notices and that the average admission is longer than 48 h, the discharge is planned ahead of time and so-called “bed blockers” are very rarely a problem. If the patient is not discharged within the given time frame the municipality must pay a daily tariff to the hospital. The primary care physician receives an electronic discharge letter written by the discharging geriatrician, detailing the in-hospital treatment, test results, health status at discharge, changes in medication, diagnoses and need for follow-up in primary care or in hospital outpatient clinics.

#### Usual care

Participants are discharged according to the procedure described above.

#### Intervention

Participants receive the same discharge procedure as the control group. In addition, an appointment is made with the participant for a follow-up visit by a geriatric nurse and a community nurse in the participant’s home. The project coordinator in charge of recruitment and randomisation calls a municipal home care coordinator when the participant has been assigned to the intervention group and discharged. The municipal home care coordinator informs the community nurse assigned to the participant, who then calls a geriatric nurse coordinator to set a date and time for the coordinated follow-up visit on a weekday within 2–5 working days after discharge. We do not expect adherence issues due to the intervention being a one-time event very close to acceptance of inclusion. Thus, we employ no strategies to improve adherence. We do not restrict any concomitant care in relation to the intervention.

At the follow-up visit, the participant, a geriatric nurse and a community nurse from the care district, to which the participant belongs, are all present. Relatives are welcome to participate and the participant is encouraged to invite them.

The geriatric nurse brings along:

- A laptop with access to the OUH electronic patient record system and a secure video channel. Medical equipment for assessing vital status (pulse oximeter, electronic sphygmomanometer, thermometer) and body weight.
- POCT technology for bed-side testing
  - Ultrasound bladder scanner (ultrasound device “Butterfly IQ + ”) [30].
  - Analyses of blood samples with immediate result
    i. HemoCue WBC DIFF Microcuvettes: Leukocytes, Neutrophiles, Lymphocytes, Monocytes, Basophiles, Eosinophiles. Capillary or venous [31].
    ii. I-STAT CHEM8 + Cartridge: Sodium (Na^+^), Potassium (K^+^), BUN/Urea, Creatinine, Haematocrit, Haemoglobin, Venous. Validated [32, 33].
    iii. QuikRead Go CRP + Hb Cartridge: CRP, Hb. Capillary [34].

At the visit, the participant, the geriatric nurse and the community nurse conducts a systematic assessment of the participant’s health and register any health-related problems. The assessment is conducted using a standardised Danish tool designed specifically for this purpose (Fælles Sprog III (FS-III) *(In English: Common Language III)*). Only a subsection of the tool, i.e. the parts covering mental well-being and bodily functions is used (“Helbredstilstande” (*In English: Health conditions*)). The tool is well known and used in the Danish home care system [35]. The Fælles Sprog III data is collected for the sole purpose of ensuring a systematic assessment.

For each item in the FS-III the geriatric nurse notes any changes in the participants’ health status, and whether the care plan has been followed, or deviations have been made. Adjustments to the care plan are made and noted accordingly. If there is any cause for concerns regarding the participant’s health compared to the time of discharge the geriatric nurse carries out vital status assessments. Further assessment by ultrasound bladder scan and/or bedside analyses of blood samples for C-reactive protein and electrolytes is based on the geriatric nurse’s clinical judgement. If the geriatric nurse finds it relevant, a geriatrician from G-OUH may be contacted by video. The geriatrician is thus able to communicate with the participant and the geriatric nurse, get the on-site clinically objective results, and finally make a clinical decision. If needed, the participant can be assessed the following day in the geriatric outpatient clinic or be acutely readmitted to the AMU.

### Randomisation procedure

The randomisation is a permuted block randomisation with block sizes 4, 6 and 8. This lowers the risk of having more follow-ups in a single week than we can manage. The permuted blocks are made to ensure that the coordinator and the staff will not be able to predict the result of the randomisation. The coordinator is not involved in the participant’s treatment or care at all, neither before nor after randomisation. We are using equivalent group distribution to maximise statistical power. There are more blocks than needed, which ensures that the final block sizes cannot be predicted.

In practice, the randomisation procedure is handled by Open Patient data Explorative Network (OPEN) [36], a research support entity also in charge of the REDCap database, a database made specifically for enrolling, randomising, data collection and data management. The allocation sequence is made by OPEN and based on a pseudo random number generator (RNG) list. It is made using software by Sealed Envelope Ltd [37], a widely used service. The list is not from a true RNG, as it has a seed^1^ to recreate the list [37]. This seed and the list, however, is unavailable to the coordinator that assigns a group to the participants (and unavailable to everyone else in the core group of this project as well—the seed is in the possession of OPEN and the list is hidden from the users involved in the project in the REDCap software). REDCap will tell the coordinator which group the participant will be in upon discharge.

### Blinding

The participant, hospital staff and project coordinator are blinded to the allocation until the participant is discharged upon which the randomisation occurs. Thus, the hospital treatment and discharge should not be affected by the enrolment of the participant. After discharge, when the patient is randomised, the participant, the project coordinator, the geriatric nurse, the community nurse, and the primary care physician will no longer be blinded. The project coordinator is not involved with the patients after the patient has been discharged, except if the patient is readmitted and invited to participate in the project again.

Prior to data analysis, all IDs will be linked to a new scrambled ID, and the old ID is hidden as will all identifying data (social security number, address). This will be done by a third party to ensure that the data analyst will not know which group is the intervention and which is the control group. All data analysis will thus be done blinded.

### Study outcome

The study outcomes are listed in the table below. Overall, we have several main focus areas:

The primary aim is to examine whether an early follow-up visit carried out simultaneously by a geriatric nurse and a community nurse can remedy a recently discharged participant’s health problems related to the hospital admission and reduce readmission. As such, the main outcome is 30 days acute readmissions. A readmission is defined as any hospital visit with a LOS of 12 h or longer, as this is in accordance with the definition of acute readmission by the Danish National Health Services [38]. Other aims include the use of in-home clinical assessment and use of POCT to understand their value in the clinical assessment, and their feasibility in an in-home setting. In addition, user perspectives on the home visit are a central outcome covering participants, community nurses, the geriatric nurses and the geriatrician. Other outcomes are the most common post-discharge health-related problems that arise or become apparent in the first days after discharge. Finally, health economic analysis will be carried out (Table 2).

**Table 2:** Project outcomes

Outcome		Outcome measure
***Primary outcomes***		
Outcome 1	Readmission (30 days)	Acute readmission to a Danish hospital within 30 days from hospital discharge
Outcome 2	Readmission (90 days)	Acute readmission to a Danish hospital within 90 days from hospital discharge
***Secondary outcomes***		
Outcome 3	Mortality (1 year)	Proportion of participants deceased within one year
Outcome 4 Outcome 5	Time to Readmission Days alive out of hospital	Proportional hazard model of readmission with a total duration of 1 year and median time to readmission estimated by Kaplan–Meier method Days spent alive out of the hospital within 30 and 90 days from discharge
Outcome 6	Clinical assessments/POCT measurements and their association to decision making	Regression analysis of the association between conducting a clinical measurement (e.g. Saturation, temperature, Sodium, CRP) or POCT analysis and the risk of readmission
Outcome 7	Costs associated with the intervention	Analysis of the costs associated with the intervention and potential savings from the result of the intervention
Outcome 8	User perspectives among involved participants and professionals	Analysis of questionnaires and focus group interviews with involved groups, including participants, community nurses, geriatric nurses, geriatric physician, and primary care physician

### Data collection

Data will be collected at several points. The data will all be saved and stored in a secure database (REDCap). All personnel involved will be trained data collection.

Before the participant is invited to join the study, data is gathered on eligibility (i.e. diagnosed moderate to severe dementia, residence location to identify citizens of Odense Municipality, and type, use of home care, terminal diseases and presence of delirium).

Before discharge, data is gathered from the participants’ electronic medical records regarding discharge diagnoses, LOS, medication, medical history and Barthel ADL score [39]. A Clinical Frailty Score [40] is assessed by a geriatric nurse.

All data collected during the follow-up visit is registered in our database, including the data gathered in the FS-III survey, vital status and POCT results. The vital parameters and POCT results will be used to understand their influence on the clinical decision making.

The data collected during the follow-up visit will be filled directly into pre-defined fields in REDCap and will help ensure that all the necessary data is collected.

All participants, controls and interventions will receive a questionnaire, which is a modified version of a previously validated questionnaire [41]. At the time of randomisation, both groups will be asked about their satisfaction with the treatment, care and hospitalisation in general, as well as if they feel safe being discharged. A follow-up questionnaire will be administered to both groups by telephone. Data will be transferred to REDCap manually. The questionnaires will be administered by project coordinators who are not involved in the clinical care of the patients.

Data on death and readmissions will be collected from electronic medical records for 1 year after the allocation date for each participant.

### Data analyses

#### Sample power and statistical power

Based on our recent finding of a 28% reduction in readmissions following geriatric follow-up to discharged geriatric patients [42], and using a 2-proportions with 2-sample equality power test, a current readmission rate of 19% (control group), and an expected 28% reduction by the intervention, the readmission rate in the intervention group is (19 − (19 × 0.28)) 13.68%. If the proportions of group A, *pA*, is (100 − 19 =) 81 and proportion of group B,* pB*, is (100 − 13.68 =) 86.32,* α* = 0.05,* β* = 0.80 and* К* = 1, the sample size should include 763 in each group, and 1526 patients totally [43]. The total finally becomes 1832 after accounting for an expected dropout rate of 20%. The Department of Geriatric Medicine at Odense University Hospital is expected to discharge about 1500–1800 patients in 2022–2023 alone, which makes it feasible to include enough participants to reach statistically significant results in 2 years. We had originally calculated a shorter time period for the study, however, due to COVID and nurse shortage, the number of beds has been reduced. We have included statistics for all inclusion and exclusion criteria in our model for estimation of the time required for the study.

### Statistical analyses

Statistical analyses will be carried out STATA Version 17 (StataCORP LLC, Texas, USA).

We will exclude participants with single-item missing data relevant to the analysis and will not perform imputations. An intention-to-treat analysis will be employed to maintain the integrity of our results. A per-protocol analysis will also be performed to complement the primary intention-to-treat analysis. While the intention-to-treat analysis preserves the advantages of randomisation and reflects real-world implementation challenges, the per-protocol analysis may help assess the true effect of receiving the intervention as intended. Given the negligible planning costs and absence of adverse effects, the intervention may still be considered clinically relevant even if statistical significance is only observed in the per-protocol population.

Characteristics will be reported with numbers, percentages and means with standard deviations or medians with interquartile range, as appropriate. Differences will be calculated using appropriate tests. Appropriate regression analyses will be conducted according to advice from a research statistician. Threshold for significance will be *P* < 0.05.

Readmission and mortality will be calculated and compared as relative risks. We will conduct an interim analysis after 1 year to adjust the time length of the study and to assure that one group does not have a significantly higher mortality rate or readmission rate than the other, which are the stopping criteria. The outcomes examined in the interim analysis will be 30-day mortality and 30-day readmission rate. The interim analysis will be conducted on data masked by a third party to ensure blinding, and the data will not be unblinded unless there is a significant difference in mortality, which is the only stopping criteria. The interim analysis will be conducted after 13 months have passed, i.e. January 2024.

The financial costs analyses will be conducted under supervision of a health economist, Professor Jørgen T. Lauridsen. We will analyse our data including time spent by the health professionals and estimate intervention cost per participant, which will be compared to an estimated daily cost of admission and the estimated avoided readmission days.

Qualitative analysis will be conducted to assess user perspectives. This will be conducted as a separate parallel study by Sanne Have Beck, RN.

We will use the EQUATOR Network guidelines, e.g. the CONSORT 2010 statement, when presenting the results of our study.

### Analyses of primary and secondary outcomes

The primary outcome is 30-day acute readmission. We will use relative risks to compare the readmission rates in the two groups. Relative risk for other readmission time frames will also be calculated to improve comparisons with other studies.

Competing risk of death will be considered in our analyses, as it can skew the results. Partially due to this, days out of hospital will also be calculated as it is less affected by the competing risk of death than a binary readmission outcome.

We will also calculate the relative risks for 30-day mortality.

A proportional hazard model will be used to explain the time to readmission. This will be done using the Cox regression model. Moreover, median time to readmission will be estimated by the Kaplan–Meier method.

We will include relevant patient characteristics that are known risk factors in our statistical analyses, e.g. multimorbidity, polypharmacy and length of stay.

Throughout the analyses, we will adjust for participants that were already readmitted at the time of recruitment. Separate analyses will be done for patients that already received the intervention, as there may be a residual effect of the intervention.

## Discussion

The future will pose a major challenge by the increasing number of older adults with complex health issues and in need of acute hospital admission. The future also implies a shortage of health care professionals in both primary and secondary care and fewer hospital beds. Preventing acute admissions and readmissions where possible is central for the well-being of the older population as well as for health care providers. Even disregarding the future challenges, preventable readmissions are of no benefit to anyone. The process of readmitting is stressful for the municipal home-based-care system, and readmissions increase pressure on the hospitals and, most importantly, have a negative impact on the patients. Preventing readmissions would be a benefit to society as a whole. Therefore, it is highly important to understand which health problems arise after discharge, especially in the first week, which is where the majority of acute readmissions occur.

The reasons for acute readmission are multiple as well as complex and mixed, and some are even unavoidable. Certain risk factors have been identified, but current tools to predict readmission (e.g., ISAR, LACE) have not yet been proven effective and further research is needed [44, 45].

Several items in transitional care are important. A list of potential issues can include poor communication between the discharging ward and the home care district resulting in lack of important information regarding the needs of care for the patient, incomplete organisation and delivery of aids, insufficient knowledge by the discharging ward of the patient’s home environment, lack of competences by the home carers, inability to recognise early signs of worsening health and delayed contact with the primary care physician after discharge. We seek to understand the factors involved in the transition from hospital to home, to intervene upon some of them and to analyse the impact of our intervention.

Previous studies using follow-up visits have shown positive effects when carried out by a geriatric team consisting of a physician and a nurse or allied health professional [14, 15]. However, as personnel resources are scarce, we aim at a cross-sectoral intervention with a trained geriatric nurse, a systematic assessment of functions (FSIII), POCT equipment for rapid clinical assessment, real-time video consultation with a geriatrician from the discharging hospital ward, and, not the least, a cross-sectoral face-to-face communication with the municipal care providers, the participants and possible relatives. This is a novel approach that, to our knowledge, has not previously been investigated. The intervention is multifactorial. As such, we will not be able to disentangle whether it is the coordinated home visit alone, the systematic approach, the clinical in-home assessment or the video consultation that is the most important. The present study is a pragmatic trial, based on the principles of Clinical Geriatric Assessment (CGA), with a holistic and cross-sectoral approach.

This study has several strengths and weaknesses. In terms of strengths, the study is a major RCT and thus it will yield a high level of evidence. The coordinator, who randomises the participants, will have no influence on treatment and care of the participants and thus no effect on readmission. The randomisation will be carried out at discharge, not during the admission, which ensures that the hospital staff is blinded and thus unbiased. As it is an RCT study, the survivorship bias will be low.

An additional strength of this study is that we also analyse the health economic aspects of the intervention, which is expected to be an important factor for potential adoption of this intervention in other clinical settings treating care needing older adults, e.g., orthopaedic departments discharging hip fracture patients. Equally important is the qualitative data from the user’s perspective.

In terms of weaknesses, there will be a selection bias towards cognitively intact or mildly cognitively impaired patients with, at most, a diagnosis of mild dementia, as patients with moderate to severe dementia will be excluded due to the current research-ethical constraints regarding the inclusion of incapacitated patients. Yet, we believe that patients with moderate to severe dementia would benefit from the intervention, too. There may also be a performance bias, as participants in the intervention group may withhold contact to primary care physician if they feel worse after discharge as they know they will receive a follow-up visit shortly. The same performance bias is expected among the home care staff who visits the participants. This type of bias may lead to lower readmissions on its own.

Finally, the intervention is multifactorial encompassing multiple elements: (a) a home visit soon after discharge that may reassure both the participant, the relatives and the home care so that contact to the primary care physician is prevented, (b) a systematic approach securing that all potential bodily functions are assessed, (c) the use of vital status assessment and POCT analyses and (d) video contact to a geriatrician from the discharging department. We will thus not be able to disentangle, which of the elements has the greatest effect in the intervention, but as geriatric patients are complex, all elements of the interventions may add valuable information that supports clinical decision-making and relevant care to the patient.

### Trial status

The study has begun on November 1st, 2022. The original date was postponed due to a severe nursing staff shortage in Denmark in the aftermath of COVID. We achieved the necessary funding and approvals to begin the study and have begun trial. The first month (November 2022) of the trial was conducted as a pilot and the data will not be included in the final data set. Difficulties include hospital policy changes that has resulted in fewer patients allocated to our ward, and a larger than expected proportion of patients that are not from the Odense Municipality. Due to these challenges, we now include readmitted patients as described earlier, as this can be adjusted in data analysis.

Protocol Version 1. Date: April 15th, 2024

Name and contact information of the trial sponsor:

Karen Andersen-Ranberg, MD, Professor, PhD

Department of Geriatric Medicine, Odense University Hospital, Odense, Denmark

J.B. Winsløwsvej 4

5000 Odense C

Denmark.

Email: karen.andersen-ranberg@rsyd.dk.

Phone: + 45 6550 3038

Role of sponsor: Not applicable

## Supplementary Information

Supplementary Material 1.

## Data Availability

Data sharing is not applicable currently. Whether data sharing will be possible after finishing the study in the future relies on obtaining permission from the Danish Data Protection Agency.

## Abbreviations

LOS: Length of stay
G-OUH: Department of Geriatrics, Odense University Hospital
POCT: Point-of-care-testing
OUH: Odense University Hospital
COPD: Chronic obstructive pulmonary disease
AMU: Acute medical unit
FS-III: Fælles Sprog III (English: Common Language III)
OPEN: Open Patient data Explorative Network
RNG: Random Number Generator
CGA: Clinical Geriatric Assessment
ADL: Activities of Daily Living
SPIRIT: Standard Protocol Items: Recommendations for Interventional Clinical Trials
CONSORT: Consolidated Standards of Reporting Trials

## Footnotes

1 A seed (or “Random seed”) is a starting point that completely determines the number sequence of a random number sequence. The seed can be used to recall the number sequence generated by a random number generator. It is thus”a key” to the number, and in this case a key to the block randomisation sequence.
